# A Polymorphism at the Translation Start Site of the Vitamin D Receptor Gene Is Associated with the Response to Anti-Osteoporotic Therapy in Postmenopausal Women from Southern Italy

**DOI:** 10.3390/ijms16035452

**Published:** 2015-03-10

**Authors:** Valeria Conti, Giusy Russomanno, Graziamaria Corbi, Giuseppe Toro, Vittorio Simeon, Walter Filippelli, Nicola Ferrara, Michela Grimaldi, Valeria D’Argenio, Nicola Maffulli, Amelia Filippelli

**Affiliations:** 1 Department of Medicine and Surgery, University of Salerno, Baronissi 84081, Italy; E-Mails: vconti@unisa.it (V.C.); afilippelli@unisa.it (A.F.); 2 Doctoral School of Translational and Clinical Medicine, University of Salerno, Baronissi 84081, Italy; E-Mail: grussomanno@unisa.it; 3 Department of Medicine and Health Sciences, University of Molise, Campobasso 86100, Italy; 4 Unit of Orthopaedics and Traumatology, “Martiri del Villa Malta” Hospital, Sarno 84087, Italy; E-Mail: giuseppe.toro@gmail.com; 5 Laboratory of Pre-clinical and Translational Research, Reference Cancer Center of Basilicata, Scientific Institute of Hospitalization and Treatment, Rionero in Vulture 85028, Italy; E-Mail: vittoriosimeon@gmail.com; 6 Department of Studies for Institutions and Territorial Systems, University “Parthenope” of Naples, Naples 80133, Italy; E-Mail: walter.filippelli@uniparthenope.it; 7 Department of Medical Translational Sciences, University of Naples Federico II, Naples 80131, Italy; E-Mail: nicola.ferrara@unina.it; 8 S. Maugeri Foundation, Scientific Institute of Telese, IRRCS, Telese Terme 82037, Italy; 9 Department of Molecular Medicine and Medical Biotechnologies, University of Naples Federico II, Naples 80131, Italy; E-Mail: dargenio@ceinge.unina.it; 10 Department of Musculoskeletal Disorders, Faculty of Medicine and Surgery, University of Salerno, Baronissi 84081, Italy; E-Mail: nmaffulli@unisa.it; 11 Centre for Sports and Exercise Medicine, Barts and the London School of Medicine and Dentistry, Mile End Hospital, 275 Bancroft Road, London E1 4DG, UK

**Keywords:** pharmacogenetics, postmenopausal osteoporosis, bisphosphonate, bone mineral density, VDR genotype, rs1544410, rs2228570

## Abstract

The present study investigated the effect of two single nucleotide polymorphisms (SNPs) of the vitamin D receptor (VDR) gene, rs1544410 A/G and rs2228570 C/T, in modulating bone mineral density (BMD) and the response to treatment with bisphosphonates or strontium ranelate in postmenopausal osteoporosis (PMO). Four hundred eighteen postmenopausal women from Southern Italy treated with bisphosphonates or strontium ranelate for three years were enrolled and stratified according to their genotype. Changes in BMD were expressed as the delta *t*-score (Δ*t*-score). Allelic frequencies for rs1544410 A/GSNP were 11.2% AA, 50.0% GA and 38.8% GG; for rs2228570 C/TSNP were 54.8% CC, 39.5% TC and 5.7% TT. TT carriers showed a lower *t*-score than TC and CC (both *p* < 0.02) genotypes and were more responsive to the therapy when compared to both TC (*p* < 0.02) and CC (*p* < 0.05) carriers. Specifically, TT carriers receiving alendronate demonstrated a significant improvement of the Δ*t*-score compared to TC and CC (both *p* < 0.0001) carriers. After adjustment for confounders, the Δ*t*-score showed evidence of a statistically significant positive association with TT in all treatments considered. Therapy response was independent of rs1544410 A/G SNP; instead, rs2228570 C/TSNP was associated with a better response to antiresorptive treatment, thus suggesting that the therapy for PMO should be personalized.

## 1. Introduction

In osteoporosis, both environmental and genetic factors are involved, producing reduced bone mineral density (BMD), an increased rate of bone loss and greater risk of spontaneous fractures. Postmenopausal osteoporosis (PMO) is a major public health problem [1]. Recently, non-invasive techniques for BMD and bone structure assessment, including dual energy X-ray absorptiometry and quantitative ultrasound, allowed studying several aspects of this disease [2,3].

BMD is generally used as the main outcome of osteoporosis genetic studies, because of its very high heritability [4]. Polymorphisms of a large number of genes are associated with variations in BMD and might play a role in predicting and possibly determining fracture risk. Several single nucleotide polymorphisms (SNPs) have been associated with osteoporotic phenotypes, such those present in vitamin D receptor (VDR), type I collagen (COLIA1) and estrogen receptor (ER) genes [5].

The polymorphic VDR gene was the first to be associated with bone turnover and bone density [6,7]. Many studies focused on SNPs located near the 3' flanking region of VDR, including rs1544410 (A/G) and rs2228570 (C/T) polymorphisms at the translational start site of the VDR protein coding sequence [8,9,10]. However, the results of these studies are not univocal.

Most anti-osteoporotic drugs act as antiresorptive agents, decreasing bone loss and increasing bone strength, and in this context, bisphosphonates represent the first-line therapy, because they show efficacy for global fracture risk reduction in addition to a good safety profile [11,12]. However, their effects vary among patients, and this can be responsible for therapy failure and the occurrence of adverse reactions [13]. More recently, the calcium mimetic, strontium ranelate, showed both antiresorptive and anabolic properties and is currently used in the management of PMO [14].

Patients show a great variability in the response to anti-osteoporotic treatments; as a consequence, management of PMO should be planned considering the relative benefits and risks in different populations and in each patient [11]. Nevertheless, few data on the pharmacogenetics of various drugs used to manage osteoporosis are available.

The present study investigated the influence of A/G and C/T VDR polymorphisms in modulating BMD and the response to therapy of osteoporosis in postmenopausal women from Southern Italy treated with bisphosphonates or strontium ranelate.

## 2. Results

### 2.1. Characteristics of the Subjects

The study population consisted of 418 postmenopausal women, defined on the basis of BMD (expressed as the *t*-score) as osteopenic (−2.5 < *t*-score < −1.5) or osteoporotic (*t*-score ≤ −2.5).

Baseline characteristics, including t-score, were reported as a function of BsmI and FokI VDR polymorphisms (Table 1). Patient subgroups were homogeneous for non-genetic factors, such as age, age at menopause, Body Mass Index (BMI), calcium intake, serum ionized calcium, alkaline phosphatase and 25-OH vitamin D. No patients had vitamin D deficiency (mean value: 33.8 ± 3.7 ng/mL).

The allelic frequencies for A/G rs1544410 SNP were 11.2% AA, 50.0% GA and 38.8% GG; for C/T rs2228570 SNP, they were 54.8% CC, 39.5% TC and 5.7% TT. While the BsmI-A/G allelic frequency was comparable to what is described in the Caucasian population [15], the FokI-C/T allelic frequency was similar to what is reported for the African-American population [16] (Table 2). The data for both SNPs were in Hardy–Weinberg equilibrium (A/G rs1544410, *p* = 0.11; C/T rs2228570, *p* = 0.52). FokI-TT carriers showed a lower t-score compared to TC and CC (both *p* < 0.02) subjects.

### 2.2. Association between Vitamin D Receptor (VDR) Genotypes and Therapy Response

To investigate the association between VDR genotypes and therapy response, we used the delta *t*-score (Δ*t*-score), calculated as the difference between the *t*-score after three years of treatment and the *t*-score at baseline. Most patients were treated with alendronate (35.9%), and at baseline, their *t*-scores were significantly higher than those of patients treated with strontium ranelate (*p* = 0.02) (Table 3). However, no significant changes between t-scores after three years of therapy and the Δ*t*-scores were found. Furthermore, no differences between serum ionized calcium and alkaline phosphatase values before and after treatments were observed. Homozygous FokI-TT carriers were better responders when compared to both TC (*p* < 0.02) and CC (*p* < 0.05) subjects, regardless of the drug used. More specifically, homozygous TT carriers treated with alendronate showed a marked improvement in term of Δ*t*-score than both heterozygous TC and homozygous CC (both *p* < 0.0001). Therapy response was independent of BsmI genotype in all patients considered (Table 4). After adjustment for potential confounders (age, age at menopause, BMI, calcium intake serum ionized calcium and alkaline phosphatase values), Δ*t*-score showed significant correlation with FokI-TT (*p* = 0.047, β = 3.374; 95% CI = 1.11–11.18), regardless of the drug considered.

Power calculation of the study was performed considering the frequency of the “T” allele equal to 0.25, the known Δ*t*-score mean value of 0.06 and SD = 0.62, with a main “expected” mean value of 0.5. Thus, a sample size of 350 subjects (with a value for α = 0.05) would produce a power of test equal to 0.836.

In the present investigation, 418 postmenopausal women were recruited; therefore, the power of the study was strong enough to evaluate the pharmacogenetic effect of FokI SNP.

## 3. Discussion

Several studies on VDR polymorphisms as factors probably involved in the regulation of bone mineral density have been performed since the original work by Morrison *et al.* [6]. Many authors reported an association between VDR genotype and BMD in postmenopausal women, but the results have been inconclusive and often contradictory.

Some studies suggested a correlation between VDR SNPs and BMD in postmenopausal women in various populations (Chinese, Indian and Maltese), while other surveys did not confirm such evidence [17,18,19].

In addition to the most investigated genetic variants spanning the 3'-end of the VDR gene (especially A/G-BsmI), also the C/T-FokI polymorphism located in the translation initiation site was shown to affect BMD. Bandrés *et al.* indicated FokI SNP as the most relevant BMD genetic determinant in postmenopausal Spanish women [9]. Falchetti *et al.* identified this SNP as an influencing factor for BMD and turnover parameters in a female population of the Italian island of Lampedusa [10]. Zajíčková *et al.*, while failing to show the influence of BsmI, ApaI and TaqI polymorphic variants on BMD, found that the presence of FokI-T allele was associated, in PMO, with a lower hip BMD [20].

The present study investigated BsmI and FokI SNPs in postmenopausal women from the Campania Region in Southern Italy, measuring the BMD at the heel by using quantitative ultrasound. We did not find a statistically significant association between BMD changes and BsmI, but such an association was instead evident with FokI.

While genetic studies on the association between VDR genotypes and bone quality have produced conflicting results, pharmacogenetic investigations on the individual response to anti-osteoporotic treatments have delivered more consistent and compelling data. Hormone replacement therapy (HRT), alone or in combination with alendronate, produces a significant increase of BMD in women after menopause [21,22,23]. Indeed, most of the pharmacogenetic studies focus on the variability of the response to HRT [3,24,25].

Although bisphosphonates are the most widely prescribed drugs for bone diseases, including PMO, very few studies focus on their pharmacogenetic aspects. Bisphosphonates share structural similarities, but show marked differences in their potency, efficacy and safety profile [12]. Alendronate, the most investigated, is safe and effective also after discontinuation of prolonged therapy and, with some other bisphosphonates, shows a persistent therapeutic effect given its long skeletal half-life [26,27,28].

Currently, data on the association between VDR genotypes and bisphosphonates, used alone or in combination in PMO, are scarce. Many authors found a relationship between BsmI and antiresorptive drug efficacy, as in the case of raloxifene and etidronate with calcium supplementation [29,30]. Moreover, A/G-BsmI influences the efficacy of antiresorptive drugs, especially when used in combination, and women bearing the G allele might be more responsive to alendronate compared to AA carriers [31,32,33]. In contrast, we found that the response to alendronate was not associated with the A/G-BsmI, but with the C/T-FokI polymorphism: women homozygous for the T allele were the best responders. Logistic regression, however, showed a statistically significant association between the presence of the T allele and the response to therapy (evaluated as the Δ*t*-score) in all treatments considered. The association between C/T-FokI VDR genotypes and Δ*t*-score remained significant also after adjustment for age, BMI, calcium intake, as well as for other potential confounders. Therefore, subjects bearing the TT genotype had lower BMD compared to those with the TC or CC genotype and were the best responders to anti-osteoporotic drugs, thus confirming the possible role played by this polymorphism in the management of PMO.

The presence of C/T-FokI SNP in exon 2 of the VDR gene generates a new start codon, implying the translation of a longer variant of VDR protein (T-VDR). Some studies suggested that the shorter variant of VDR protein (the wild type, C-VDR) exhibits greater transcriptional activity [34]. By contrast, other studies did not find functional differences between T-VDR and C-VDR [35]. Therefore, a consensus has not been achieved. It is possible that in our study population, in addition to FokI SNPs, other factors, both genetic and environmental, concur to determine the therapeutically favourable response of ff (TT) carriers.

To our knowledge, no data are available on the association between C/T-FokI and the response to anti-osteoporotic treatments in postmenopausal women. In the present study, we showed for the first time the association between the T allele of such an SNP and both BMD changes and variability in response to therapy, highlighting that C/T-FokI genotyping could be important in the management of osteoporosis on an individual basis. Compared to bisphosphonates, many studies have indicated strontium ranelate, which has both antiresorptive (anti-catabolic) and anabolic properties, as a promising treatment for PMO [36,37], but based on the data presented in this study, we were not able to confirm this evidence.

The genetic makeup of patients influences their response to pharmacological therapy. Genetic variability widely impacts the homeostasis of the body [38], and as a consequence, a variety of pharmacogenetic studies are now accumulating to personalize the clinical management of several diseases [39], osteoporosis being no exception [40]. Osteoporosis is also a major socio-economic burden on health service resources; thus, improvement in the prevention and management of this condition has a societal impact. Most pharmacogenetics studies focused on A/G-BsmI impact on anti-osteoporotic therapy response variability in postmenopausal women [33]. The present investigation suggests the involvement of the C/T-FokI VDR polymorphism in determining the effects of antiresorptive treatment in PMO, underlining the importance of the association between genetic variants and the response to different drugs. This observational study allowed us to compare the outcomes of different anti-osteoporotic treatments on the basis of the patients’ genetic background.

It must be stressed that, in the present study, the TT-FokI allelic frequency was different from that described in the Caucasian population, but similar to what was found in some non-Caucasian populations, such as African-American and Maltese [16,19]. Moreover, we found that the frequency of this allele was also different from what was reported by Gennari *et al.* in postmenopausal women from Tuscany, Italy [41]. To avoid misclassification of genotypes because of technical problems, we genotyped 10% randomly selected samples by direct sequencing: this confirmed the results obtained by the restriction fragment length polymorphism (RFLP) method.

Indeed, in a genome-wide analysis of 126K autosomal SNPs within the Italian population, Di Gaetano *et al.* demonstrated that the genetic structure of the Italian population was strongly influenced by geographical location [42]. Northern Italians were genetically close to the French population, whereas Southern Italians had some similarities with other Mediterranean populations, such as those from the Middle East. In addition, North African ancestry is highest in South West Europe [43]. However, further studies in a larger cohort of individuals should be undertaken to confirm these findings. The present work has some limitations, including the fact that it was not possible to control vitamin D changes in all patients; in fact, this control was performed only on the serum of 42 randomly selected patients due to limited funds available for this research. However, all enrolled patients at baseline showed vitamin D serum level mean values of 33.8 ± 3.7 ng/mL, and according to the International Osteoporosis foundation, which recommends a desirable 25-OH serum level of 30 ng/mL [44], they had no vitamin D deficiency; moreover, in the 42 randomly-selected patients, no changes occurred in vitamin D levels (mean values of 32.3 ± 3.6 ng/mL at three-year follow up; *p* = no significant).

**Table 1:** Characteristics of all subjects and according to the rs1544410 and rs2228570 genotypes.

Variables	All	rs1544410				rs2228570			
		AA	AG	GG	*p*	CC	CT	TT	*p*
*n*	418	47	209	162		229	165	24	
Percentage	100	11.2	50.0	38.8		54.8	39.5	5.7	
Age (years)	66.56 ± 8.63	62.35 ± 7.80	66.81 ± 8.45	65.26 ± 8.41	0.13	66.86 ± 8.39	66.02 ± 8.77	67.20 ± 12.57	0.81
Age at menopause (years)	47.90 ± 5.60	48.64 ± 6.44	48.72 ± 5.66	47.79 ± 6.47	0.77	47.86 ± 5.65	48.40 ± 5.48	48.63 ± 5.50	0.82
Weight (kg)	67.97 ± 10.93	67.69 ± 12.06	68.76 ± 10.57	66.61 ± 12.31	0.70	68.10 ± 10.61	68.71 ± 11.98	68.20 ± 11.26	0.96
Height (cm)	158.49 ± 5.68	158.92 ± 7.67	159.52 ± 5.41	156.97 ± 5.19	0.14	158.72 ± 5.68	159.13 ± 5.85	155.80 ± 5.63	0.48
BMI (kg/m^2^)	27.04 ± 4.00	26.69 ± 3.7	27.02 ± 3.92	27.02 ± 4.64	0.97	27.03 ± 3.82	27.09 ± 4.41	28.03 ± 4.02	0.86
Ca^2+^ (mg/day)	1210.44 ± 20.55	1216.1 ± 23.59	1209.13 ± 20.64	1211.89 ± 20.32	0.39	1212.99 ± 22.63	1207.73 ± 17.76	1212.2 ± 20.67	0.24
BMD (*t*-score)	−2.11 ± 0.66	−2.21 ± 0.66	−2.05 ± 0.61	−2.12 ± 0.75	0.69	−2.12 ± 0.63	−2.06 ± 0.64	−2.68 ± 0.51 ^a^	0.017
Ca^2+^ serum (mmol/L)	9.68 ± 0.66	9.78 ± 0.66	9.75 ± 0.78	9.63 ± 0.45	0.88	9.63 ± 0.67	9.79 ± 0.65	10.45 ± 0.49	0.24
APs (UI/L)	179.30 ± 51.40	192.33 ± 34.40	185.05 ± 53.02	147.60 ± 39.67	0.09	174.42 ± 52.09	194.86 ± 17.86	175.00 ± 94.75	0.62
Vitamin D (ng/mL)	33.83 ± 3.69	34.02 ± 3.88	33.91 ± 3.74	33.65 ± 3.61	0.91	33.94 ± 3.73	33.95 ± 3.83	33.53 ± 3.38	0.97

Values are the mean ± standard deviation; The *p*-value was calculated by the ANOVA test; BMI, body mass index; Ca^2+^, calcium intake; BMD, bone mineral density; Ca^2+^ serum, serum ionized calcium; APs, serum alkaline phosphatase; vitamin D, serum 25 OH vitamin D; The frequency of rs1544410 and rs2228570 alleles in postmenopausal women was in Hardy–Weinberg equilibrium (*p* = 0.11 and *p* = 0.52, respectively); ^a^ Significantly lower than CC and CT genotypes (both *p* < 0.02) by Bonferroni’s test.

**Table 2:** Distribution of rs2228570 genotypes in different populations.

Population	Allelic Frequency		
	CC (%)	CT (%)	TT (%)
Central Italy ^a^	41	45	14
Southern Italy (Campania)	54.8	39.5	5.7
Maltese ^b^	60.4	30.7	8.9
African-American ^c^	46	48	6

The frequency of the T-allele in the study population was similar to that described in Maltese and African-American populations; ^a^ Data from Gennari *et al.* [41]; ^b^ Data from Vidal *et al.* [19]; ^c^ Data from Zmuda *et al.* [16].

**Table 3:** Therapy response.

Variables	All	Ibandronate	Risedronate	Alendronate	Strontium Ranelate	*p*
*n* subjects (%)	418 (100)	53 (12.7)	115 (27.5)	150 (35.9)	100 (25.4)	
*t*-score at baseline	−2.11 ± 0.66	−2.00 ± 0.56	−2.18 ± 0.72	−1.97 ± 0.74 ^a^	−2.31 ± 0.40	0.02
*t*-score after therapy	−2.05 ± 0.72	−1.98 ± 0.82	−2.12 ± 0.65	−1.96 ± 0.74	−2.22 ± 0.65	0.19
Δ*t*-score	0.06 ± 0.62	0.04 ± 0.57	0.06 ± 0.45	0.03 ± 0.77	0.05 ± 0.48	0.99
Ca^2+^ serum (mmol/L) at baseline	9.68 ± 0.66	9.25 ± 0.61	9.87 ± 0.82	9.60 ± 0.46	9.47 ± 0.74	0.41
Ca^2+^ serum (mmol/L) after therapy	9.62 ± 0.77	9.00 ± 0.53	9.93 ± 0.84	9.49 ± 0.69	9.53 ± 0.84	0.20
APs (UI/L) at baseline	179.30 ± 51.40	168.00 ± 32.81	170.75 ± 33.41	171.53 ± 64.89	183.37 ± 41.25	0.38
APs (UI/L) after therapy	176.44 ± 46.78	164.25 ± 34.76	169.17 ± 29.15	170.47 ± 61.70	181.27 ± 40.70	0.31

Values are the mean ± standard deviation; The *p*-value was calculated by the ANOVA test; Ca^2+^ serum, serum ionized calcium; APs, serum alkaline phosphatase; ^a^ Significantly higher than strontium ranelate (*p* = 0.02) by Bonferroni’s test; Serum ionized calcium before and after therapy (ns); Serum alkaline phosphatase before and after therapy (ns).

**Table 4:** Therapies according to rs1544410 and rs2228570 genotypes.

Variables	rs1544410				rs2228570			
	AA	AG	GG	*p*	CC	CT	TT	*p*
All therapies (%)	47 (11.2)	209 (50.0)	162 (38.8)		229 (54.8)	165 (39.5)	24 (5.7)	
*t*-score at baseline	−2.21 ± 0.66	−2.05 ± 0.61	−2.12 ± 0.75	0.65	−2.12 ± 0.63	−2.06 ± 0.64	−2.68 ± 0.51 ^b^	0.017
*t*-score after therapy	−2.23 ± 0.66	−2.07 ± 0.58	−2.05 ± 0.86	0.69	−2.04 ± 0.71	−2.07 ± 0.60	−2.08 ± 1.15	0.94
Δ*t*-score	−0.03 ± 0.30	0.01 ± 0.40	0.05 ± 0.82	0.89	0.11 ± 0.66	−0.02 ± 0.41	0.60 ± 1.29 ^c^	0.013
Ibandronate (%)	9 (2.1)	22 (5.3)	22 (5.3)		31 (7.4)	22 (5.3)	0 (0)	
*t*-score at baseline	−2.43 ± 0.93	−1.84 ± 0.39	−2.06 ± 0.71	0.42	−2.19 ± 0.46	−1.76 ± 0.33	−	0.02
*t*-score after therapy	−2.53 ± 0.68	−2.17 ± 0.51	−2.11 ± 0.72	0.63	−2.02 ± 0.99	−1.94 ± 0.45	−	0.84
Δ*t*-score	−0.07 ± 0.55	−0.32 ± 0.26	−0.06 ± 0.38	0.43	0.22 ± 0.73	−0.19 ± 0.38	−	0.15
Risedronate (%)	19 (4.5)	43 (10.3)	53 (12.7)		79 (18.9)	26 (6.2)	10 (2.4)	
*t*-score at baseline	−2.33 ± 0.65	−2.14 ± 0.64	−2.28 ± 0.67	0.78	−2.19 ± 0.61	−2.45 ± 0.68	−2.62 ± 0.70	0.30
*t*-score after therapy	−2.27 ± 0.70	−1.96 ± 0.68	−2.25 ± 0.53	0.39	−2.10 ± 0.60	−2.36 ± 0.71	−2.57 ± 0.64	0.26
Δ*t*-score	0.02 ± 0.29	0.18 ± 0.52	0.06 ± 0.49	0.71	0.11 ± 0.52	0.16 ± 0.32	0.05 ± 0.26	0.90
Alendronate (%)	19 (4.5)	78 (18.7)	53 (12.7)		64 (15.3)	79 (18.9)	7 (1.7)	
*t*-score at baseline	−1.98 ± 0.60	−1.81 ± 0.71	−1.92 ± 0.98	0.86	−1.92 ± 0.80	−1.89 ± 0.68	−2.87 ± 0.59	0.09
*t*-score after therapy	−1.98 ± 0.64	−1.97 ± 0.66	−1.91 ± 1.08	0.97	−1.97 ± 0.62	−1.93 ± 0.64	−0.93 ± 1.59 ^d^	0.048
Δ*t*-score	−0.06 ± 0.19	−0.05 ± 0.38	−0.07 ± 1.22	0.99	0.03 ± 0.81	−0.07 ± 0.44	1.94 ± 1.87 ^e^	0.0001
Stronzium ranelate (%)	0 (0)	66 (15.8)	34 (8.1)		55 (13.2)	38 (9.1)	7 (1.7)	
*t*-score at baseline	–	−2.33 ± 0.36 ^a^	−2.22 ± 0.42	0.42	−2.27 ± 0.42	−2.32 ± 0.47	−2.57 ± 0.11	0.52
*t*-score after therapy	–	−2.26 ± 0.36	−1.88 ± 1.05	0.18	−2.07 ± 0.82	−2.27 ± 0.44	−2.57 ± 0.23	0.41
Δ*t*-score	–	0.04 ± 0.27	0.26 ± 0.81	0.31	0.14 ± 0.59	0.06 ± 0.35	0.00 ± 0.35	0.87
*p*-value								
*t*-score at baseline	0.57	0.022	0.54		0.18	0.05	0.78	
*t*-score after therapy	0.54	0.38	0.65		0.92	0.12	0.11	
Δ*t*-score	0.90	0.07	0.78		0.87	0.19	0.08	

Values are the mean ± standard deviation; The *p*-value was calculated by analysis of variance (ANOVA) with Bonferroni’s *post hoc* test; ^a^ Significantly lower than alendronate (*p* = 0.02); ^b^ Significantly lower than CC (*p* < 0.05) and CT (*p* < 0.02) genotypes; ^c^ Significantly higher than CC (*p* < 0.05) and CT (*p* < 0.02) genotypes; ^d^ Significantly higher than CC genotype (*p* < 0.05); ^e^ Significantly higher than CC and CT genotypes (both *p* < 0.0001). After adjustment for potential confounders (age, age at menopause, BMI, calcium intake, serum ionized calcium and alkaline phosphatase values), the Δ*t*-score showed a significant correlation with rs2228570 TT (*p* = 0.047, β = 3.374; 95% CI = 1.11–11.18).

## 4. Experimental Section

### 4.1. Subjects

The study population included 500 postmenopausal women from the Campania Region, Italy, treated with anti-osteoporotic drugs, consecutively referred at the Unit of Orthopedics and Traumatology of Hospital “Martiri del Villa Malta” in Sarno, Salerno (Italy), for osteoporosis screening.

Exclusion criteria were represented by the presence of chronic diseases, known syndromes potentially interfering with bone metabolism or blood coagulation (such as pituitary diseases, hyperthyroidism, primary hyperparathyroidism) or drug therapy, including corticosteroids, hormone replacement therapy or warfarin. Women treated with calcium and vitamin D supplements were also excluded from the study. 

A total of 418 postmenopausal women were eventually included in the study. The medical history and information about the calcium dietary intake and physical activity of the subjects were recorded according to a self-reporting questionnaire [45]. Patients’ information and consent forms were approved by the ethical committee of ASL of Salerno Registry of Observational Studies (RSO) n.12/11. The study was conducted in accordance with the World Medical Association’s 2008 Declaration of Helsinki. This report adheres to the consolidated standards for the reporting of observational trials and was written according to the Strengthening the Reporting of Observational Studies in Epidemiology (STROBE) guidelines for Observational Studies in Epidemiology.

All patients underwent a quantitative ultra-sound (QUS) test at the heel using a Hologic Sahara instrument (Hologic Inc., Bedford, MA, USA). QUS allows estimating BMD (eBMD) measuring bone status indices, such as broadband ultrasound attenuation (BUA), speed of sound (SOS) and the Quantitative Ultrasound Index (QUI). BMD values were expressed as the *t*-score, which indicates the number of standard deviations above or below the mean of ideal BMD value for a healthy 30-year-old adult of the same sex and ethnicity as the patient [46].

Of the 418 postmenopausal women included in the study, 312 were treated with bisphosphonates (alendronate, risedronate and ibandronate) and 106 with strontium ranelate.

We used the delta *t*-score (Δ*t*-score, calculated as the difference between the *t*-score after three years of therapy and the *t*-score at baseline) to evaluate the association between VDR genotypes and the therapy response.

Blood samples were collected after overnight fasting, and serum was stored at −80 °C until analyses. Aliquots (500 μL) of whole blood (EDTA-stabilized blood) were used for A/G-BsmI and C/T-FokI SNPs analysis. Serum levels of 25-OH vitamin D were measured by an ELISA kit (DRG International, Inc., Springfield Township, NJ, USA) in all patients at baseline and in 42 randomly-selected patients at the 3-year follow up.

### 4.2. VDR Genotyping

We performed the analysis of the rs1544410 and rs2228570 polymorphisms of the VDR gene using the restriction fragment length polymorphism (RFLP) technique. Genomic DNA was extracted from EDTA-stabilized blood using a commercially available DNA extraction kit (Qiagen, Hilden, Germany). For rs1544410 genotyping, we performed PCR in a final volume of 30 μL containing 100 ng of genomic DNA as the template. The forward primer was 5'-CAACCAAGACTACAAGTACCGCGTC-3', and the reverse primer was 5'-AACCAAGCGGAAGAGGTCAAGGG-3'. PCR conditions were as follows: initial denaturation at 94 °C for 5 min, then 35 cycles of denaturation for 60 s at 94 °C, annealing for 60 s at 58 °C and extension for 60 s at 72 °C. The reaction was terminated by 7-min elongation at 72 °C. The PCR amplification product was then digested with the BsmI restriction enzyme (New England Biolabs Inc., Ipswich, MA, USA) at 65 °C for 2 h; the resulting fragments were separated on 3% agarose gel. The presence of the BsmI site was indicated as G, while its absence was designated as A.

For C/Trs2228570 genotyping, we performed PCR in a 30 μL mixture containing 100 ng of genomic DNA as the template. The forward primer was 5'-TGGGTGGCACCAAGGATG-3', and the reverse primer was 5'-CCTTCATGGAAACACCTTGC-3'. PCR conditions were as follows: initial denaturation at 94 °C for 5 min, then 35 cycles of denaturation for 30 s at 94 °C, annealing for 30 s at 58 °C and extension for 30 s at 72 °C. The reaction was terminated by 7-min elongation at 72 °C. The PCR product was digested with the FokI restriction enzyme (New England Biolabs Inc., Beverly, MA, USA) at 37 °C for 3 h; the resulting restriction fragments were visualized on a 3% agarose gel. The presence of the FokI restriction site was indicated as T, while its absence was designated as C.

### 4.3. Statistical Analysis

SNPs were tested for deviations from Hardy–Weinberg equilibrium using the exact test in PLINK Version 1.07 software (http://pngu.mgh.harvard.edu/purcell/plink/) [47].

Differences between multiple groups were evaluated by analysis of variance (ANOVA) with the Bonferroni *post hoc* test and are presented as the means ± SD. *p* < 0.05 was considered as statistically significant for all analyses.

A multivariate analysis was performed to assess correlations among variables. All analyses were carried out using the Statistical Package for the Social Sciences (SPSS 15.0, SPSS Inc., Chicago, IL, USA). The analysis of statistical power calculation was performed using the Quanto program (Version 1.2.4; University of Southern California, Los Angeles, CA, USA).

## 5. Conclusions

In conclusion, we suggest that C/T-FokI SNP, but not A/G-BsmI SNP, may influence the antiresorptive treatment response in postmenopausal osteoporosis, thus supporting the concept that therapy should be individualized. However, further studies should be performed in a larger cohort of postmenopausal women in Southern Italy to assess the actual impact of VDR genotypes on both BMD and anti-osteoporotic therapies response.
